# The long-term impact of infant rearing background on the affective state of adult common marmosets (*Callithrix jacchus*)

**DOI:** 10.1016/j.applanim.2015.10.009

**Published:** 2016-01

**Authors:** Hayley Ash, Hannah M. Buchanan-Smith

**Affiliations:** Behaviour and Evolution Research Group and Scottish Primate Research Group, Psychology, School of Natural Sciences, University of Stirling, Scotland, United Kingdom

**Keywords:** Animal welfare, Affective state, Rearing, Cognitive bias, Anhedonia, Marmosets

## Abstract

Early life environment, including temporary family separation, can have a major influence on affective state. Using a battery of tests, the current study compared the performance of adult common marmosets (*Callithrix jacchus*), reared as infants under 3 different conditions: family-reared twins, family-reared animals from triplet litters where only 2 remain (2stays) and supplementary fed triplets. No significant differences were found in latency to approach and obtain food from a human or a novel object between rearing conditions, suggesting no effect on neophobia. There were no differences in cognitive bias task acquisition time, or proportion of responses to each ambiguous probe. Very minor differences were found in response to the probes, with only supplementary fed marmosets making fewer responses to the middle probe, compared to the probe nearest the rewarded stimuli. Similarly, in a test for anhedonia, no difference was found between rearing conditions in consumption of milkshake at different concentrations. There was just one very small difference in reward motivation, with only supplementary fed triplets demonstrating a lack of preference for milkshake over water at the lowest concentration. This consistent pattern of results suggests that the supplementary feeding of large litters of marmosets at this facility did not have a major effect on welfare, and is unlikely to influence performance in reward-related scientific tasks. Therefore, while family separation is not recommended, this particular practice should be used if it is necessary, such as to reduce infant mortality. Regular positive interactions with humans are also encouraged, to reduce fear and improve welfare of marmosets kept in captivity.

## Highlights

• Family-reared and supplementary fed marmosets were compared in adulthood.
• Temperament, cognitive bias and preference tests were used.
• Very few differences were found between rearing backgrounds.
• The supplementary feeding practice does not have a substantial impact on welfare.
• The practice described should be used if supplementary feeding is necessary.

## 1 Introduction

The common marmoset is characterised by twin births, and the care of all members of the family during infancy, which is known to be important for their development (Dettling et al., 2007). However, triplets are increasingly common in captive colonies of laboratory-housed marmosets (*Callithrix jacchus*), with larger litter sizes associated with higher infant mortality (Ash and Buchanan-Smith, 2014) and perinatal stress (Reiss, 1996). Human intervention is therefore practiced when families have litters larger than two, to improve infant survival. Partial hand-rearing can be performed, in which either one or all infants are removed from the family for certain periods of the day, or complete hand-rearing, involving permanent family absence, can be carried out. Early separation from the family could however induce changes in cognition and behaviour, increasing anxiety or depression-like symptoms. In human populations, adverse experiences in childhood can also increase the risk of developing mood disorders (Parker and Maestripieri, 2011). Rearing methods are therefore an important issue in captive primate care, although husbandry practices are often advocated without sound scientific evidence of their success (Buchanan-Smith, 2010a).

Previous work has used parental separation paradigms, to look at response to novelty later in life. Maternally deprived primates were significantly more neophobic, showing greater behavioural disturbance and less exploration in a novel environment (Spencer-Booth and Hinde, 1971: rhesus macaque, *Macaca mulatta:* Caine et al., 1983: pigtail macaque, *Macaca nemestrina*), and peer-reared primates have been found to display hyperemotional behaviour (Capitanio, 1986: *Macaca nemestrina*), as well as reduced responsiveness (Capitanio et al., 2005: *Macaca mulatta*), which could indicate differences in anxiety and depression. Paul et al. (2000) found that maternal deprivation led to anhedonia-like states in adult rhesus macaques, using two-bottle choice tests. All animals drank more sweet solution than water, although there was a reduced preference in those maternally deprived, compared to controls. As well as this, bonnet macaques (*Macaca radiata*) with mothers exposed to unpredictable foraging demands showed reduced sociability as adults (Rosenblum and Andrews, 1994), possibly reflecting an anhedonic state (Pryce et al., 2005).

Pryce et al. (2005) studied the effects of daily unpredictable isolation from parents as infants in common marmosets. They removed the infants from their natal group each day and placed them alone in a cage, for variable durations and times, from post-natal days 2 to 28. While early deprived (ED) marmosets performed similarly to controls on a simple discrimination task, they made significantly more errors following visual reversal. ED animals may therefore be unable to respond flexibly to environmental change. ED marmosets were also found to perform significantly fewer progressive ratio operant responses (Pryce et al., 2004), indicating a diminished response to rewarding stimuli.

It is therefore possible that stressful early life events could alter long-term mood states. Cognitive bias, described as the propensity of an individual to exhibit behaviour indicating anticipation of either a relatively positive or negative outcome, in response to affectively ambiguous stimuli, has recently emerged as a promising tool for the assessment of emotion in animals (Mendl and Paul, 2004). Negative cognitive biases are reliable indicators of distress in humans, and are implicated in affective disorders. There is also accumulating research demonstrating cognitive biases in animals, including dogs, *Canis lupus familiaris* (Mendl et al., 2010), rats, *Rattus norvegicus* (Burman et al., 2008), European starlings, *Sturnus vulgaris* (Matheson et al., 2008), sheep, *Ovis aries* (Doyle et al., 2010), honeybees, *Apis mellifera carnica* (Bateson et al., 2011) and rhesus macaques (Bethell et al., 2012). Results have demonstrated that animals develop a more negative outlook following a stressful event, and a more positive outlook following a positive event, such as enrichment. Early life family separations could therefore increase the perception of threats in the environment (Prinz, 2004), inducing negative cognitive biases indicative of depression or anxiety (Eysenck et al., 1987).

However, there is some evidence that hand-reared animals are less anxious and fearful when exposed to later challenges (Pryce et al., 2003: *Rattus norvegicus*), with no difference in learning speed, accuracy or perseveration, compared to mother-reared animals (Feenders and Bateson, 2013: *Sturnus vulgaris*). These results suggest emotionally driven decision making was altered in a way generally associated with reduced developmental stress (Feenders and Bateson, 2013). Parker et al. (2004) also used the parental separation paradigm in squirrel monkeys (*Saimiri sciureus)*, exposing them to weekly 1 h ‘stress inoculation’ separations from the natal group, for 10 weeks at 17 weeks of age. Following subsequent exposure to a novel environment, they displayed less maternal clinging, as well as more exploration and food consumption, suggesting they were less anxious than non-‘stress-inoculated’ monkeys.

It is therefore important for ethical and scientific reasons to understand the psychological consequences of hand-rearing practices (Bethell et al., 2012), as rearing background could have an impact on welfare, as well as introduce unwanted variability in scientific output, obscuring treatment effects and increasing the number of animals needed (Howard, 2002). Although variations of the parental separation model are commonly practiced in colonies of marmosets bred for use in research and testing, features of such early life stress, including type, duration and frequency, as well as degree of deprivation, can all play a part in producing diverse developmental outcomes (Parker and Maestripieri, 2011). It is possible that, as marmosets are adapted to being transferred between carriers from a young age (Ingram, 1977), separation from the family with litter mates, as well as predictable timing of separation and positive experiences with humans, may all minimise potential stress. Cognitive bias, as well as responses in preference and temperament tests, may be useful in assessing such effects on affective state in marmosets raised under different backgrounds.

The aim of this study was to establish the impact of rearing background on a battery of tests reflecting learning and affective state in adult common marmosets. Supplementary fed animals, exposed to early life family separations, were compared to undisturbed family-reared animals. Each subject was first given a human interaction and novel object test. A ‘Go/No Go’ cognitive bias task was then developed for use with captive marmosets. Time to learn the task, as well as response to ambiguous probes, was measured. Cognitive bias testing was followed by a two bottle choice test, to measure anhedonia. It was predicted that early separated marmosets would display greater latencies to approach novel objects and humans, as well as display a more negative cognitive bias and a reduced interest in an appetitive liquid, compared to family-reared marmosets, if this practice mimics primate parental separation paradigms (e.g. Pryce et al., 2005).

## 2 Method

### 2.1 Study animals

Twenty five adult common marmosets, housed at Dstl, Porton Down, UK, were studied (aged between 1 year 4 months and 3 years 1 month). All animals were purpose-bred in captivity. Each marmoset was housed in vasectomised male mixed-sex pairs, as stock animals (generally from approximately 20 months old, following a period of same sex housing after weaning from the natal group at 18 months). One animal per pair was studied. Their partner was randomly allocated from available animals at the time of pairing, and so was not often of the same background.

Marmosets were studied from three rearing conditions, based upon practices carried out at the breeding facility, and so no manipulations were used. Condition 1 had eight family-reared twins (4 males, 4 females). Condition 2 had nine family-reared marmosets from triplet litters where only 2 remain, due to loss of the third (known as 2stays) (4 males, 5 females), to examine potential differences in born litter size. Condition 3 had eight supplementary fed triplets (full description below) (5 male, 3 female), to examine differences in rearing background. One twin female failed to learn the cognitive bias visual discrimination task within the time period of 8 weeks, and so was not tested, although results were included from the novel object, human interaction and preference tests. Methods were approved after review by the Stirling University Psychology Ethics Committee and the facility involved.

#### 2.1.1 Supplementary-feeding

On postnatal day 1, the family member carrying the infant/s was encouraged towards the front of the home cage with a piece of marshmallow, so restraint was not necessary, and the infant/s removed from their back. All three infants were taken out of the family group together, wrapped in towelling and placed in an incubator. The litter was removed from the family daily for 2 × 2 h (8:00–10:00 am, 16:00–18:00 pm). Each infant was given SMA milk, and handled for approximately 5 min while they were fed. They received four feeds per day until they were 20 days old (0.5 ml of milk/feed at 1 week, 1–1.5 ml at 2 weeks and 1–2.5 ml at 3–4 weeks). This was reduced to three feeds, with 1 × 2 h morning session in the incubator and one afternoon feed, after which they were immediately returned to the family, until they were 25 days old. After this age, there was no incubator time, with feeds reduced to two a day between 26 and 30 days old, and to one a day between 31 and 41 days old (up to 5 ml of milk after 4 weeks). For the remainder of their time they were left with the family.

#### 2.1.2 Husbandry of all infants

Infants from all rearing conditions were removed from their family group at day 10 for weighing, and subsequently every month when each marmoset in the room was weighed. For the remainder of their time they were left with the family. All animals received a human socialisation programme, which involved the technicians offering food to the whole family and sitting in the home cage with them. The marmosets were also trained to accept banana milkshake from a syringe. Both husbandry practices were carried out once a week.

#### 2.1.3 Housing and husbandry of adults

The experiment was conducted on adult pairs of marmosets, which were housed in cages measuring 100 cm wide × 60 cm deep × 180 cm high, lined with wood chippings and furnished with a nestbox, wooden platforms, perches, ropes, suspended toys and a wire veranda. There were 3 stock rooms, each containing between 10 and 18 pairs. All marmosets had ad libitum access to water, and food was delivered twice a day. Primate pellets were fed in the morning, and a variety of fruit was provided in the afternoon. This was supplemented with malt loaf, egg, rusk, mealworms, dates, peanuts and bread on alternating days. Gum arabic and milkshake (with added Vitamin D once a week) were also given twice a week, and a constant supply of forage mix was available. Enrichment was introduced once a week, where paper parcels, cardboard boxes or bottles were provided with forage mixed into sawdust. Temperature and humidity were at 23–24 °C and 55 ± 10% respectively. Lighting was provided on a 12 h light/dark cycle, with a dawn and dusk phase.

### 2.2 Temperament tests

#### 2.2.1 Response to novel object

The novel object test was conducted first, to prevent the marmosets from being influenced by an experimenter who had previously given them food (Bowell, 2010). Two plastic film canisters (one for each animal, to prevent one individual dominating the food source) were filled with pieces of chopped banana, as this is a favoured food (Caldwell et al., 2009), increasing motivation to obtain the reward. It also has a strong aroma, ensuring the marmosets were able to detect the presence of food. The pots were placed face down on a shelf, the door shut and a stopwatch started. The observer stood in front of an adjacent cage and avoided looking directly at the test cage, which can be threatening for marmosets. Latency from closing the door to when the test subject first touched the canister and when they first obtained the banana was recorded. A time limit of 2 min (120 s) was imposed (based on Bowell, 2010).

#### 2.2.2 Response to human interaction

The novel object test was followed by the human interaction test, on the same day. Both tests were carried out between 9:00 and 11:00, after the animals had their morning feed. The experimenter approached the marmosets’ home cage slowly, at an angle of approximately 45°. Standing approximately 30 cm away, without facing directly into the cage or looking at the marmosets, two pieces of dried papaya or pineapple (a favoured food as indicated by preference tests with non-study marmosets) was offered, one for each animal. Latency to take the reward from the hand was recorded, up to 2 min (based on Bowell, 2010). If the study animal's partner dominated the food source, or appeared to prevent the test subject from approaching, they were distracted by providing another piece of food lower in the cage, while the reward was offered to the study animal in the original position. The non-test animal was never rewarded in the test animal's location.

### 2.3 Habituation for cognitive bias and preference testing

All training and testing was conducted in the home environment, to avoid potential confounds with separation, and neither food nor fluid management was employed. The marmoset was first allowed 2 days to familiarise themselves with the new apparatus. They were then enclosed in the veranda on the front of the home cage, to allow individual testing (Pryce et al., 2004). Sessions were carried out once a day between 9:00 and 12:00. If there was more than 10 s of persistent escape attempts at any time, the animal was allowed to leave immediately, although this was rare. At the end of each daily session, the monkey was rewarded with a favoured piece of dried fruit.

### 2.4 Cognitive bias

#### 2.4.1 Apparatus

**Fig. 1:**
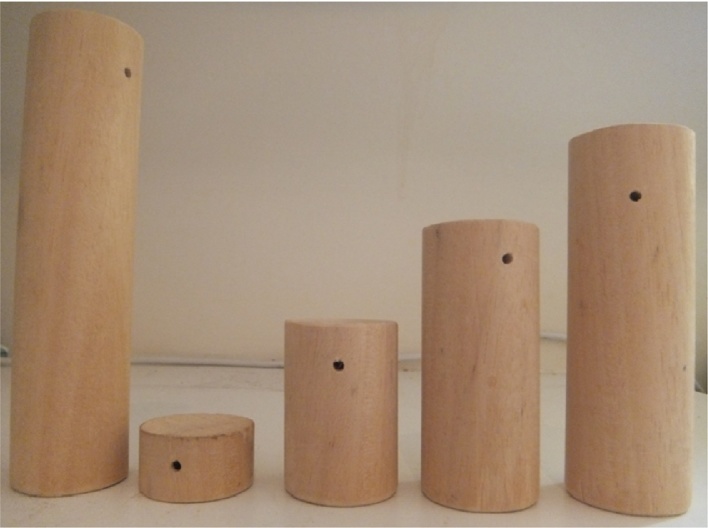
Cognitive bias stimuli and probes.

**Fig. 2:**
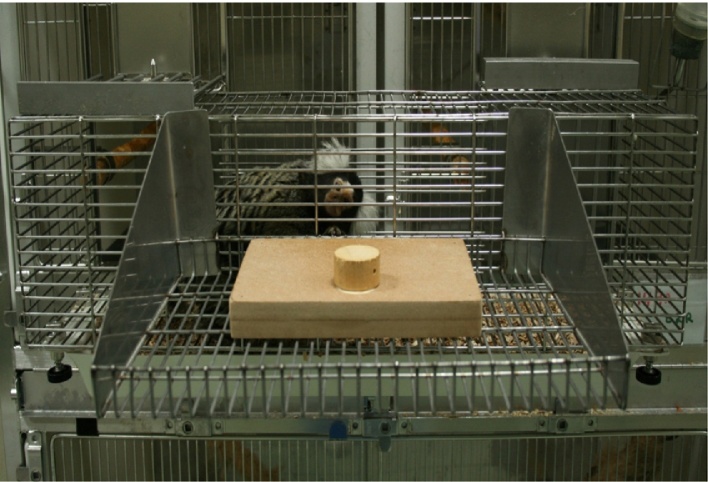
Cognitive bias apparatus set-up in the home cage.

A visual discrimination task was employed, in which a single tube was presented outside the veranda, on a tray attached to the front of the cage. Reference stimulus tubes (S+ and S−) were 2 cm and 15 cm in height. Three unreinforced ambiguous probe heights were evenly distributed at intermediate points between the two reference heights: one located midway (PI) between the reference points, while the other two (P+ and P−) were halfway between the central probe and each reference height (11.5 cm, 8.5 cm, 5.5 cm) (based on Bethell et al., 2012). Fig. 1 shows the cognitive bias stimulus and probes. Fig. 2 shows the set-up of the apparatus in the animal's home cage. A small piece of rusk was hidden under each stimulus (both S+, S− and probes), to prevent olfactory cues. Half of the animals were allocated the largest tube as the reinforced stimulus, while the other half were allocated the smallest tube, to counterbalance the rewarded and unrewarded conditions (Bethell et al., 2012).

#### 2.4.2 Training

‘Go/No go’ task training sessions were conducted, in which single stimuli were presented (Burman et al., 2008). Correct ‘Go’ responses to S+ were rewarded with an accessible treat (rusk was revealed for access on a 100% fixed ratio schedule). Correct ‘No go’ responses to S− were unrewarded (inaccessible treat, in which rusk was not revealed for access, with a 2-s inter-trial interval), while incorrect ‘Go’ responses were followed by a 5-s time-out punishment (following Pryce et al., 2004). The number of trials taken to achieve criterion was recorded, to look at any differences between conditions in training performance (Mendl et al., 2009). There were three stages of training, to shape the behaviour gradually. All sessions lasted for 5 min maximum, or if the marmoset earned the maximum amount of rewards (22 pieces). The training schedule was as follows:

##### 2.4.2.1 Stage A (rewarded)

The marmoset was presented with the rewarded height and encouraged to touch it to obtain the reward (following Pryce et al., 2004). A 5-s time limit was imposed for responses, with a maximum of 20 trials. A new trial began when the animal either received the reward or 5 s had passed with no response. They were considered trained when the animal was calmly moving around the enclosed space, reliably touching the tube and taking the reward for 80% of presentations, over 3 consecutive days. Stage B then began.

##### 2.4.2.2 Stage B (fixed rewarded and unrewarded)

The unrewarded height was introduced. In trials 1–22, the rewarded height was presented for two consecutive trials, the unrewarded height for the next two trials and this process repeated (Burman et al., 2008). The first and last trials were always rewarded, to maintain interest in the task. A 2-s response time was imposed, with a new trial starting if there was no response within this period. This presentation time was selected, as it allowed enough time for the animals to respond on ‘Go’ trials and process the food reward, while ensuring that attention was maintained during ‘No go’ trials. This session continued until the animal responded correctly on 80% S+ trials and 80% S− trials, over 3 consecutive days, before Stage C commences.

##### 2.4.2.3 Stage C (random rewarded and unrewarded)

A pseudorandom schedule was then used, with the 20 training entries divided between rewarded and unrewarded heights. No more than two rewarded or unrewarded heights occurred consecutively, and equal numbers of both were presented (Burman et al., 2008). The first and last trials were always rewarded. A 2-s response time was imposed. Training was considered completed when the animal was responding correctly on 80% S+ trials and 80% S− trials (Bethell et al., 2012), over 3 consecutive days.

#### 2.4.3 Cognitive bias testing

Twenty trials were carried out during each test session. Three unreinforced ambiguous height trials (probes, P+, PI, P−) were interspersed, on trials 6, 12 and 18. The overall sequence alternated between rewarded and unrewarded heights, starting and finishing with a rewarded trial. There was the same number of ambiguous trials following a rewarded height as an unrewarded height. The presentation order was counterbalanced over 3 test days, with heights depending on the learned S+ and S−. The number of ‘Go/No go’ responses to ambiguous heights were recorded. A pseudorandom training day was presented between the test days, to re-establish the learnt discrimination task and ensure the animals were performing to criterion (based on Bethell et al., 2012). Only cognitive bias sessions where correct responses were made on at least 80% of trained stimuli were included, to ensure that attention was being maintained throughout the session. Occasionally subjects were distracted, failing to meet this criterion, and so these sessions were omitted.

### 2.5 Reward motivation: two bottle preference tests

After completion of cognitive bias testing, monkeys were not tested for 1 week (following Pryce et al., 2004). Reward motivation was then assessed, using a two bottle preference test. A pilot study was first conducted, to confirm the marmosets’ significant preference for milkshake over water. Once a day, the animals were allowed access to the testing box for 2 min, to drink from a pair of identical 60 ml drinking bottles. These were simultaneously presented in the middle of the veranda (following Laska, 1997). One bottle contained tap water, and the other contained one of four concentrations of Nesquik banana milkshake (60 ml water with 1 scoop of powder; ¾, ½ and ¼ scoops). There were therefore 4 trials, over 4 separate days. Bottle positions were alternated daily to control for position preference, and concentration pairs were counterbalanced between animals to avoid order effects. Consumption of water and milkshake was measured at the end of access (Paul et al., 2000). As there was no significant association between body weight and amount of fluid consumed over the test days, data were analysed in ml consumed, rather than ml/g.

### 2.6 Statistical analysis

Data were first checked for underlying assumptions of normality, using the Kolmogorov–Smirnov test. Where no transformation was successful in making data normally distributed, non-parametric tests were conducted. Kruskal–Wallis tests were used to look at differences between rearing conditions in latency to retrieve and obtain food in the human interaction and novel object tests. Mann Whitney tests were also conducted to look at differences between genders within rearing conditions.

As data were normally distributed, a 2 way ANOVA (between rearing condition × between gender) was used to look at differences in cognitive bias task acquisition time. Using Cochran's *Q* tests (an extension of Kruskal–Wallis test, for dichotomous data), no significant difference of testing day was found on proportion of responses made to each probe, in any rearing condition, and so data were collapsed across the testing sessions. The proportion of ‘Go’ responses was calculated over the three test days (sum of responses/number of days). Kruskal–Wallis tests were used to examine differences between rearing conditions in response to each probe. Friedman tests were conducted to look at within rearing condition differences in response to each probe, with follow up Wilcoxon tests. Mann Whitney tests were used to look at gender differences within rearing conditions.

A mixed factor 3 × 2 ANOVA (between rearing condition × between gender) was conducted to investigate differences in consumption of milkshake at each concentration. Despite transformations, water consumption data within the rearing conditions remained non-normal. Wilcoxon tests were therefore used to look at differences between milkshake and water consumption at each concentration within each rearing condition. Although a number of tests were carried out, adjustments were not made for multiple comparisons, so as not to increase the risk of Type II error and to allow independent assessment of the validity of results.

## 3 Results

### 3.1 Temperament tests: novel object and human interaction tests

**Fig. 3:**
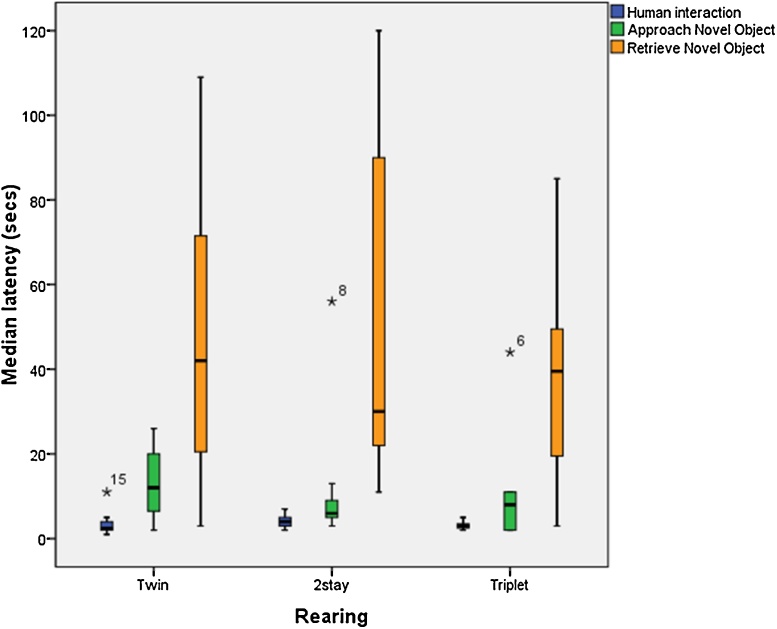
Median latencies (s) to obtain food from a human, and to approach and retrieve food from the novel object in each rearing condition. Median: solid line; Interquartile range: boxes; Minimum and Maximum value: whiskers; Outliers: stars.

Results showed that 100% of animals approached the novel object, with 96% obtaining the food. There was no significant difference between the rearing conditions in latency to approach or obtain food from the novel object. In the human interaction test, 100% of the animals took food from the hand well within the 2-min time limit. There was no significant difference between rearing conditions in latency to take food from a human. There was no within rearing condition effect of gender in either test. Fig. 3 displays the median latencies to approach and obtain food in the human interaction and novel object tests in each rearing condition.

### 3.2 Cognitive bias

#### 3.2.1 Effect of rearing condition on task acquisition time

**Fig. 4:**
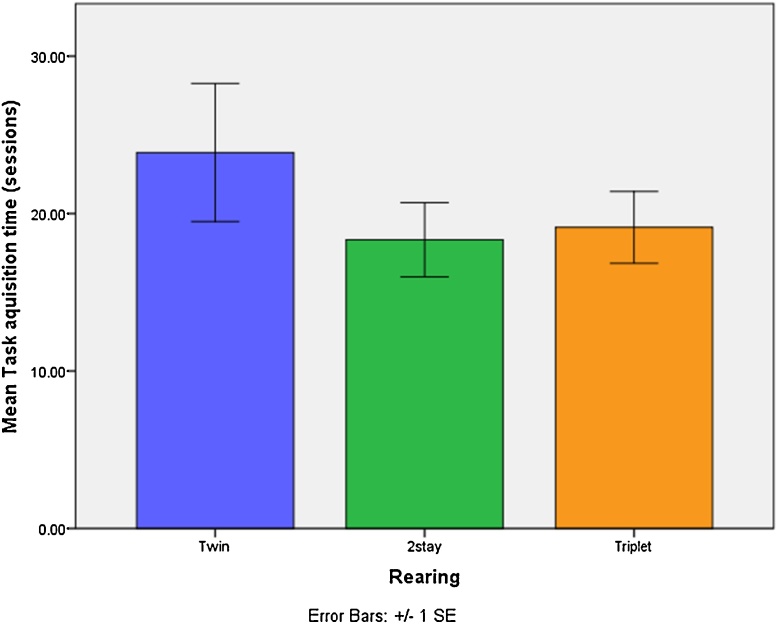
Mean number of sessions taken to complete training for the cognitive bias visual discrimination task in each rearing condition.

It took a mean of 20.36 ± 8.93 training sessions to learn the task. Many learnt in less than 20 sessions (4 weeks), although some took the full 40 sessions (8 weeks). A ceiling value of 40 was used for the one individual that didn’t learn. There was no significant effect of rearing condition or gender in time taken to complete the visual discrimination training. Fig. 4 shows the mean number of sessions taken for each rearing condition to complete the cognitive bias training task.

#### 3.2.2 Effect of rearing condition on response to probes

No significant difference was found between the rearing conditions in ‘Go’ responses to P+, PI or P−. Variation in responses to PI and P− was large, particularly in twins. No differences were found between males and females in any rearing condition for any probe.

#### 3.2.3 Response to probes within rearing conditions

**Fig. 5:**
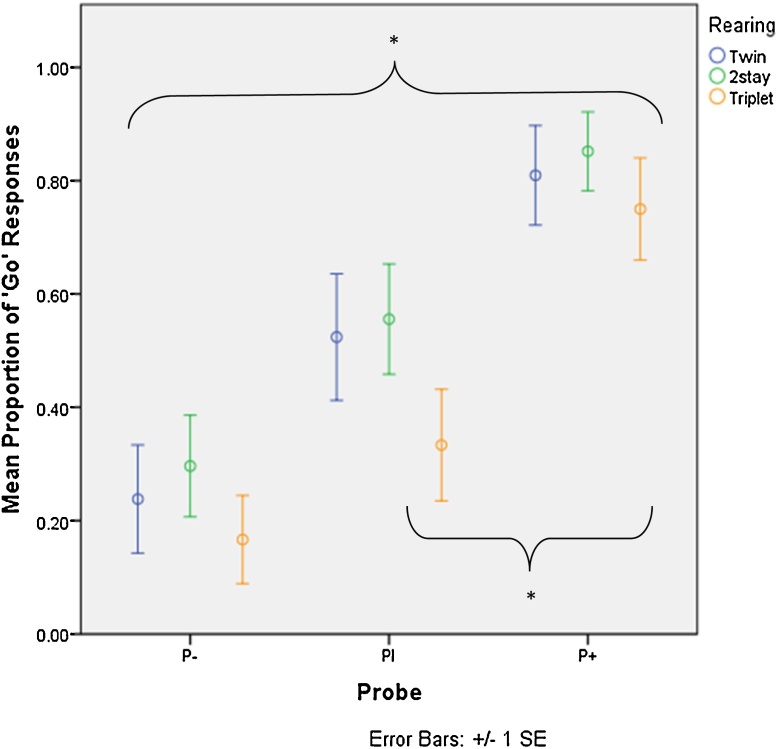
Mean proportion of responses (some medians = 0) on probe trials (P+, PI, P−) for twin, 2stay and supplementary fed triplet marmosets (*p < 0.05).

For twins, there was a significant difference in the proportion of ‘Go’ responses to each probe (Friedman test: *X*^2^_2_ = 11.00, *P* = 0.004), with significantly more responses to P+ than P− (Wilcoxon test: *Z* = −2.39, *P* = 0.017). For 2stays, there was a significant difference in the proportion of ‘Go’ responses to each probe (*X*^2^_2_ = 9.85, *P* = 0.007), with significantly more responses to P+ than P− (*Z* = −2.54, *P* = 0.011). There was also a significant difference in proportion of ‘Go’ responses to each probe for supplementary fed triplets (*X*^2^_2_ = 11.12, *P* = 0.004). There were significantly more responses to P+ than PI (*Z* = −2.54, *P* = 0.011), as well as more responses to P+ than P−. (*Z* = −2.03, *P* = 0.042). Fig. 5 displays the mean proportion of ‘Go’ responses to each probe in each rearing condition.

### 3.3 Preference tests

#### 3.3.1 Effect of rearing condition on milkshake consumption

There was no significant effect of rearing, gender or rearing*gender interaction in amount of milkshake consumed at the 0.25, 0.50 or 0.75 concentrations. There was no main effect of rearing or gender at the 1.00 concentration. There was a significant rearing*gender interaction at this concentration, with twin males drinking less than twin females, and supplementary fed triplet females drinking less than supplementary fed triplet males (ANOVA: *F*(2) = 3.619, *P* = 0.047).

#### 3.3.2 Preferences within rearing conditions

**Fig. 6:**
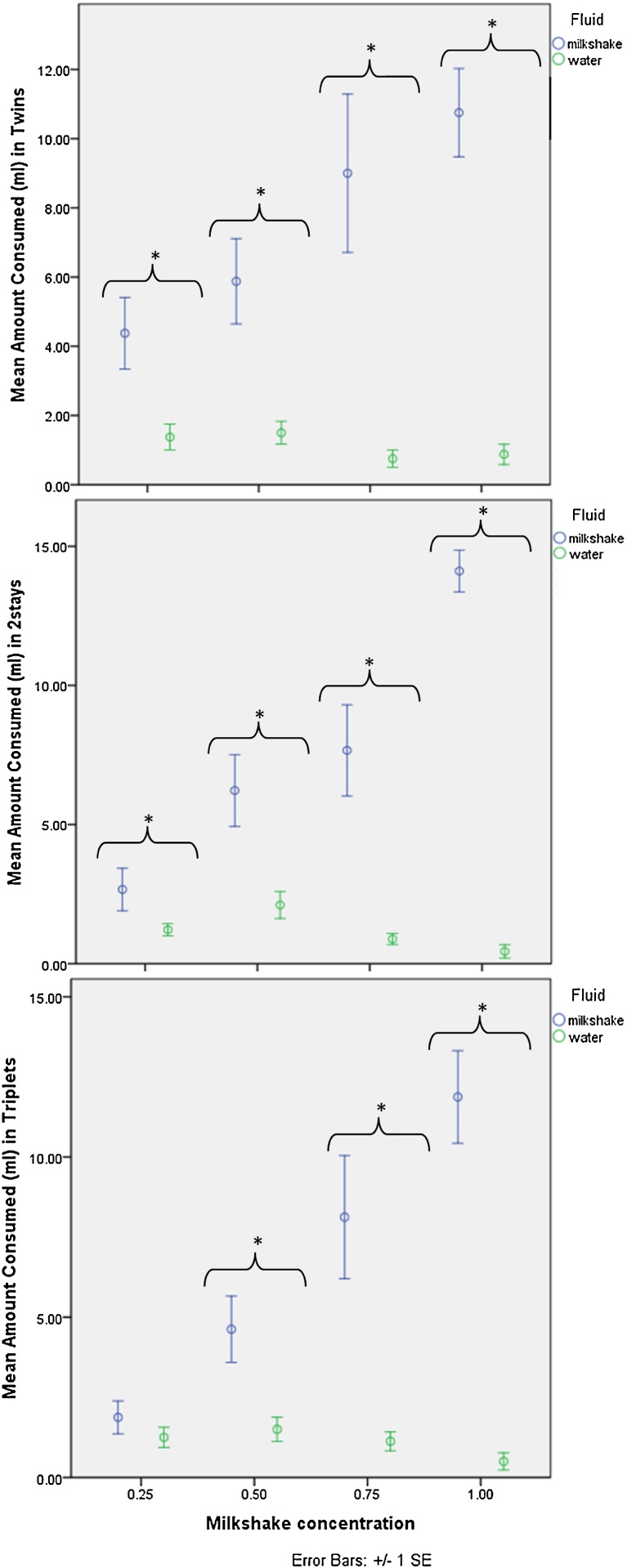
(a–c) Mean amount of milkshake and water consumed (ml) at each milkshake concentration for twin, 2stay and supplementary fed triplet marmosets (*p < 0.05).

In twins, significantly more milkshake was consumed than water at 0.25 (Wilcoxon: *Z* = −2.38, *P* = 0.018), 0.50 (Z = 2.38, P = 0.018), 0.75 (*Z* = −2.53, *P* = 0.012) and 1.00 (*Z* = −2.313, *P* = 0.021). Fig. 6a shows the amount of water and milkshake consumed at each concentration for twin marmosets. In 2stays, significantly more milkshake was consumed than water at 0.25 (*Z* = −2.03, *P* = 0.042), 0.50 (*Z* = −2.53, *P* = 0.012), 0.75 (*Z* = −2.55, *P* = 0.011) and 1.00 (*Z* = −2.67, *P* = 0.008). Fig. 6b shows the amount of water and milkshake consumed at each concentration for 2stay marmosets. In supplementary fed triplets, milkshake was consumed significantly more than water at 0.50 (*Z* = −1.97, *P* = 0.049), 0.75 (*Z* = −2.53, *P* = 0.012) and 1.00 (*Z* = −2.52, *P* = 0.012), but not at 0.25. Fig. 6c shows the amount of water and milkshake consumed at each concentration for supplementary fed triplet marmosets.

## 4 Discussion

It was hypothesised, based on numerous primate models, that early family separation would have adverse developmental consequences, including learning impairments and depressive-like symptoms (Parker and Maestripieri, 2011). However, there were very little differences in measures of learning, reward motivation and affective state between marmosets of different litter sizes and rearing backgrounds, suggesting no adverse consequences in animals born as triplets or receiving supplementary feeding during infancy, compared to family-reared twins. The results therefore demonstrate the success of this particular supplementary feeding practice in minimising any negative welfare consequences of early life family separation.

### 4.1 Temperament tests

While some previous work has found increased neophobia in primates separated from the family early in life (Spencer-Booth and Hinde, 1971: *Macaca mulatta*; Caine et al., 1983: *Macaca nemestrina*), other work has found hand-rearing led to less neophobic (Feenders and Bateson, 2013: *Sturnus vulgaria*) and less anxious animals (Parker et al., 2004: *Saimiri sciureus).* Differences between studies may however be due to the severity of the procedure used or the species investigated, with macaques often being very maternally bonded. There was no evidence that supplementary fed triplets in the current study were more fearful than family-reared marmosets. There was in fact no difference between animals raised under different conditions in time taken to retrieve food from an unknown human, or in latency to approach and obtain food from a novel object.

All animals quickly accepted food from the hand, within 3–4 s, which is an encouraging finding, suggesting they are not fearful of humans. All animals studied also approached the novel object, with 96% accessing the food. The results are in contrast to Bowell (2010), who found that only 80% of marmosets (n = 30) were willing to touch the novel object, with 47% obtaining the food, and two thirds taking food from the hand. These findings suggest differences in husbandry between the facilities, with the present colony receiving more regular human socialisation.

### 4.2 Cognitive bias

Few studies have looked at the effect of separation from the family on behavioural responses in depression-related tests, with many focusing on HPA and monoamine effects (Pryce et al., 2005). After learning a ‘Go/No Go’ task, the response to intermediate probes was used to quantify cognitive bias. Task acquisition took an average of 20 sessions, with no significant difference between rearing conditions. While other studies have suggested a link between early life stress and impaired learning in primate species, there was no evidence for this in the current study. Results are instead similar to work by Feenders and Bateson (2013), who found no difference in cognitive ability between hand-reared and family-reared starlings. Pryce et al. (2004) also found no difference in learning a simple discrimination task, although impairments in ED common marmosets were evident following reversal.

There were also no significant differences in response to each probe between the rearing conditions, although there were some small differences when each condition was analysed separately. For twins and 2stays, there were significantly more responses to P+ than P−. However, in supplementary fed triplets, there were significantly more responses to P+ than both PI and P−. Although the supplementary fed triplets may have learnt that the probes did not lead to a reward, no significant differences in response were found over the test days. Animals that were separated from their family during infancy could therefore have reduced the probability of receiving the worst outcome (a time out) by refraining from touching the most ambiguous probe. Similarly, Pryce et al. (2004) also found ED marmosets to be more sensitive to loss of control with respect to rewarding events. However, the data suggest there were only minor differences between family-reared and supplementary fed marmosets.

Despite this, there were large variations in responses to probes between individuals, suggesting cognitive biases did emerge. Such variation could be due to probes following S+ verses S− trials. As rusk was highly favoured, animals with a positive bias may continue to respond following S+, and also increase the chance of reward by responding following S−. However, animals with a negative bias may fail to respond after S+, as they have had their guaranteed reward, and loose attention following S−. As all marmosets continued to respond to the trained stimuli, differences in response to ambiguous probes were not due to reduced general activity or attention (Bateson et al., 2011). However, this does not mean these processes involve the conscious experience of emotion (Bateson et al., 2011). Alternative explanations may include differences in arousal, motivation and risk taking (Bethell et al., 2012).

### 4.3 Preference tests

Reduced consumption of appetitive food or drink have been found in choice tests and progressive ratio tests, as a marker of reward systems. Similarly to the cognitive bias tests, there were no differences between rearing conditions in consumption of each milkshake concentration, and only subtle differences in preference found when each condition was examined separately. A significantly greater amount of milkshake was consumed than water at each concentration in twins and 2stays, while in supplementary fed triplets there was only no preference for milkshake over water at the 0.25 concentration. These marmosets were therefore mildly less interested in reward at the lowest concentration, consistent with the mildly reduced expectation of reward in the cognitive bias tests.

Previous preference test studies have however found more striking anhedonic-like states in maternally deprived primates, such as Paul et al. (2000). Pryce et al. (2004) also found reduced motivation to obtain reward in ED common marmosets. Results are more similar to other work, particularly in rats, which have found no differences in appetitive fluid consumption between maternally separated individuals, compared to non-handled or early handled individuals (Crnic et al., 1981).

### 4.4 Effect of separation from the family

Although research into maternal deprivation has found severe long-term effects (Pryce et al., 2005), supplementary fed triplets in the current study displayed little differences in affective state, compared to family-reared marmosets. One major difference between this study and previous family separation studies in primates is that the infants were in continuous contact with their litter mates, and so were not isolated during their time away from the family. As well as this, the marmosets were fed at predictable times, building positive experiences with humans and novel situations from an early age, and are naturally adapted to being passed between carriers (e.g. Ingram, 1977), which could mean they are less stressed during separation than other primate species. The separations were also brief, with infants in the natal group for 20 h of the day, and reintegrated back into the family completely by 8 weeks old.

Increased duration or severity of deprivation would likely have led to greater differences between supplementary fed and family-reared marmosets (Parker and Maestripieri, 2011). For example, practices that have involved isolating young marmosets for long periods of time have led to significant adverse effects (Pryce et al., 2005). Therefore, other commonly used rearing practices, including partial and particularly complete human hand-rearing, could be a major source of stress and undermine an individual's ability to cope with challenges. However, Parker and Maestripieri (2011) suggest that overcoming moderate stress in early life, such as perhaps in the supplementary feeding practice studied, could actually increase resilience.

The lack of any meaningful differences between rearing conditions in the current study could also be due to the ongoing socialisation and training programmes that all the animals receive throughout their life at the colony. Primates in laboratories have been found to benefit greatly from socialisation with humans, starting early in life (Tasker, 2012). Regular positive interactions are associated with a reduction in anxiety related behaviours (Bassett et al., 2003), and fear responses to novel humans and situations later in life (JWGR, 2009). These are therefore important, practical husbandry Refinements. Socialisation takes little time and training, making routine implementation cheap and easy to fit around daily husbandry routines. All staff can participate in the simple task of hand-feeding their animals, which can reduce fear and improve the welfare of large numbers of captive primates (Rennie and Buchanan-Smith, 2006).

The current supplementary feeding procedure, along with a regular human socialisation programme, therefore appears to minimise the potential stress and adverse welfare effects of early family separation, and should be used if human intervention is necessary. As marmosets are widely used as models in biomedical research, the lack of major differences between rearing conditions could also mean that unwanted variability in scientific output is kept to a minimum, which would help to reduce the number of animals needed in research and testing.

Future research could look at behavioural and physiological responses to challenges, which have been more commonly investigated. It may also be beneficial to examine the effects of potentially more stressful hand-rearing practices, or those used to raise marmosets in zoos and as ‘pets’, as results may be specific to the laboratory. As welfare involves the personal experience of individual animals (Fraser, 2008), concerns raised apply to all those breeding marmosets.

## 5 Conclusion

The present study investigated whether rearing background had a long-term effect on learning, reward motivation and affective state in common marmosets. No major differences in adulthood were found across rearing conditions, with supplementary fed triplets showing only very minor reductions in expectation of and interest in reward, compared to family-reared marmosets. The current study is therefore useful in demonstrating that supplementary feeding had no long-term negative welfare consequences, at least following the practice at the colony studied. Despite the success of the current method for rearing triplets, separation from the family is not recommended, if it is possible to keep the infants with the natal group (JWGR, 2009). Appropriate housing and husbandry (Buchanan-Smith, 2010b), as well as regular positive human interactions, are encouraged for the effective management of captive animals. These will allow monkeys to become more resilient to the laboratory environment, and avoid fear as a scientific confound.
